# A Spiking Neural Network Model of Rodent Head Direction Calibrated With Landmark Free Learning

**DOI:** 10.3389/fnbot.2022.867019

**Published:** 2022-05-26

**Authors:** Rachael Stentiford, Thomas C. Knowles, Martin J. Pearson

**Affiliations:** Bristol Robotics Laboratory, University of the West England Bristol, Bristol, United Kingdom

**Keywords:** spiking neural network, pyNEST, head direction, predictive coding, localization, continuous attractor

## Abstract

Maintaining a stable estimate of head direction requires both self-motion (idiothetic) information and environmental (allothetic) anchoring. In unfamiliar or dark environments idiothetic drive can maintain a rough estimate of heading but is subject to inaccuracy, visual information is required to stabilize the head direction estimate. When learning to associate visual scenes with head angle, animals do not have access to the ‘ground truth' of their head direction, and must use egocentrically derived imprecise head direction estimates. We use both discriminative and generative methods of visual processing to learn these associations without extracting explicit landmarks from a natural visual scene, finding all are sufficiently capable at providing a corrective signal. Further, we present a spiking continuous attractor model of head direction (SNN), which when driven by idiothetic input is subject to drift. We show that head direction predictions made by the chosen model-free visual learning algorithms can correct for drift, even when trained on a small training set of estimated head angles self-generated by the SNN. We validate this model against experimental work by reproducing cue rotation experiments which demonstrate visual control of the head direction signal.

## 1. Introduction

As we move through the world we see, touch, smell, taste and hear the environment around us. We also experience a sense of our own self-motion through our vestibular system, which enables us to keep balance and to maintain an internal estimate of our location and heading—pose—in the world. Any drift in pose estimate incurred through the integration of self-motion cues alone (as we walk with our eyes closed for example) is quickly corrected when we open our eyes and recognize familiar features in the environment. This approach to self-localization has been adopted in many fields of engineering that require an accurate and persistent pose estimate to operate effectively, such as mobile robots and augmented reality devices.

The relative contributions of self-motion (idiothetic) and external sensory cues (allothetic) to the firing properties of ‘spatial cells' in rodents has been extensively investigated in neuroscience (see below for review). The integration of idiothetic cues provides the animal with a rapid and constant estimate of pose. This estimate not only aids navigation in the absence of allothetic cues, but is also a learning scaffold to associate pose with novel visual scenes. This second function has received little attention in prior models which often use the ground truth pose of a learning mobile agent to associate sensory view rather than the drift prone estimate provided from idiothetic cues. In mobile robotics this problem is addressed in the research field known as Simultaneous Localization And Mapping (SLAM) with myriad solutions proposed, each with their own advantages and limitations based on environmental, sensory and computational constraints. In this study we are interested in modeling how this problem has been solved in the mammalian brain.

The problem of accumulative error in idiothetic cue integration implies that head direction estimates from early explorations of a novel environment should be more reliable than later explorations. Therefore, earlier experiences of an environment should be used to learn associations between heading and visual scenes, to correct for drift as the animal later explores the same environment. From a learning perspective this puts constraints on the size and richness of training sets. This bi-directional learning problem is investigated here through a series of controlled experiments using a simulated mobile robot within a virtual environment. Our contribution is to model the head direction cell system using populations of spiking neurons, translating angular head velocity cues into a spike encoded representation of head direction. To anchor head directions to allothetic cues, we have trained three different model-free visual learning algorithms: Convolutional Neural Network (CNN), Variational Auto-Encoder (VAE) and Predictive Coding Network (PCN), to associate distal natural visual scenes with the spike based representation of head direction. We demonstrate that all three models are capable of correcting the drift in pose estimate from purely idiothetic cue integration even when trained on small self-generated training sets. This is evaluated further through cue conflict experiments to reveal similar characteristics of the model performance as recorded in rodents. The primary motivation for this work is toward a deeper understanding of the complete head direction system of the rodent through the integration of components modeled at different levels of abstraction; namely, spiking neural attractor network models, deep learning based generative and discriminative models, and simulated robotic embodiment. However, the integration of models at multiple levels of abstraction also provides a framework for how energy efficient neuromorphic hardware components (Krichmar et al., 2019) can be usefully integrated into mobile robotic applications in the near future. We contend that to fully exploit this biologically inspired computing paradigm requires continued biomimetic study of fundamental neuroscience as epitomized in the field of neurorobotics.

### 1.1. Rodent Head Direction Cell System

Neural correlates of position (O'Keefe, 1976; Hafting et al., 2005), environmental boundaries (Lever et al., 2009), heading (Taube et al., 1990), speed (Kropff et al., 2015) and numerous other spatial measures (see Grieves and Jeffery, 2017 for review) have been extensively studied in the rodent brain and remain an active topic in neuroscience research. Of these, head direction (HD) cells—cells which exhibit high firing rates only in small arcs of head angle—appear simplest, and have been a popular target for modeling. Head direction cells have also been identified in regions homologous to the rodent hippocampus in birds (Ben-Yishay et al., 2021), fish (Vinepinsky et al., 2020), and insects (Kim et al., 2017). Strikingly in Drosophila these cells are arranged as a ring in the Ellipsoid Body, and have properties of a continuous attractor.

Most models of head direction use a continuous attractor, where a sustained bump of activity centered on the current heading is formed and maintained through interactions between excitatory and inhibitory cells. Many rely on recurrent excitatory collaterals between cells in the Lateral Mammillary Nuclei (LMN; Zhang, 1996; Page and Jeffery, 2018), however anatomical data show no evidence of this type of connection (Boucheny et al., 2005). Although head direction cells have been found in many brain regions, including Anterior Thalamic Nuclei (ATN; Taube, 1995), Retrosplenial cortex (Cho and Sharp, 2001), Lateral Mammillary nuclei (LMN; Stackman and Taube, 1998) and Dorsal Tegmental Nucleus (DTN; Sharp et al., 2001; see Yoder et al., 2011 for review), generation of the head direction signal is thought to be in the reciprocal connection between LMN and DTN (Blair et al., 1999; Bassett and Taube, 2001). As the DTN sends mainly inhibitory connections to the LMN, attractor networks exploiting connections between two populations of cells appear more biologically plausible (Boucheny et al., 2005; Song and Wang, 2005).

### 1.2. Control of HD by Self-Motion Cues

Self-motion cues can be derived directly from the vestibular system but also from optic flow and motor efference copy. Disrupting vestibular input to head direction cells abolishes spatial firing characteristics and impacts behaviors which rely on heading (Yoder and Taube, 2009, 2014). Cells sensitive to Angular Head Velocity (AHV) have been recorded in several regions including the DTN (Bassett and Taube, 2001; Sharp et al., 2001). These cells are either sensitive to AHV in a single direction (clockwise or anticlockwise; asymmetric AHV cells) or the magnitude of AHV regardless of direction (symmetric AHV cells). Methods of moving the bump of activity on the ring attractor to follow head movement rely mainly on asymmetric AHV input. Bump movement is achieved either through imbalance between two populations of cells in the attractor network (Boucheny et al., 2005; Bicanski and Burgess, 2016), or via conjunctive cells which fire strongly as a function of both AHV and head direction (Sharp et al., 2001; McNaughton et al., 2006). However, using imprecise self-motion cues in the absence of vision results in drift in the preferred firing direction of head direction cells (Stackman et al., 2003).

### 1.3. Visual Control of HD

Although HD cells still show some directional sensitivity in the absence of visual cues or novel environments (Goodridge and Taube, 1995; Taube and Burton, 1995; Goodridge et al., 1998; Stackman et al., 2003), vision is clearly an important factor for stabilizing the head direction system. During development, head direction cells have much sharper tuning curves after eye opening (Tan et al., 2015), but may use other types of allothetic information, such as tactile exploration of corners of the environment with whiskers, to stabilize head direction before eye opening (Bassett et al., 2018). Even in unfamiliar environments, visual information helps to stabilize head direction, suggesting ongoing learning of visual landmarks (Yoder et al., 2011). In familiar environments, the preferred firing directions of head direction cells become entrained to visual features and will follow these cues over self-motion signals (Taube and Burton, 1995). When environmental cues are rotated, preferred firing directions of many cells also rotate through the same angle, resulting in “bilateral” preferred firing directions; Page and Jeffery (2018) suggest these bilateral cells may be useful for assessing the stability of environmental landmarks. Some head direction cells won't follow these big conflicts in cue location, suggesting multiple populations of head direction cells that are more or less strongly controlled by allothetic input (Dudchenko et al., 2019). This visual control of head direction begins at the LMN (Yoder et al., 2015), stabilizing the head direction signal at its origin. Both the postsubiculum (PoS) and retrosplenial cortex (RSC) are likely candidates for delivering this visual information to the LMN (Taube et al., 1990; Vann et al., 2009). Head directions cells (or compass neurons) have also been shown to follow visual information in Drosophila (Fisher et al., 2019). In this case visual inputs onto compass neurons are inhibitory, and plasticity between cells encoding visual features and compass cells has been directly observed (Kim et al., 2019).

There are two main methods of using visual information to control the head direction bump position. The first is to use visual information to fine tune the model of AHV through a learning mechanism, whether that be by detecting error between the estimated head angle and the expected head angle based on the visual cue (Kreiser et al., 2020), or using a combination of strategic behavior and landmark tracking to match the AHV model to the movement of the cue within the visual field (Stratton et al., 2011). The second method is to influence the head direction bump position directly by exploiting the attractor dynamics and injecting current into the new bump position. This could simply use Gaussian inputs into the ring attractor at determined positions (Song and Wang, 2005), or by representing features in multiple “landmark bearing cells,” learning the association between head angle and visible features, and feeding back expected head direction onto the head direction cells (Yan et al., 2021). A combination of these two methods of visual control is probably the answer to accounting for environmental or body changes in the real world; in this study we begin with directly influencing the bump position.

### 1.4. Models of Visual Input

**Figure 1:**
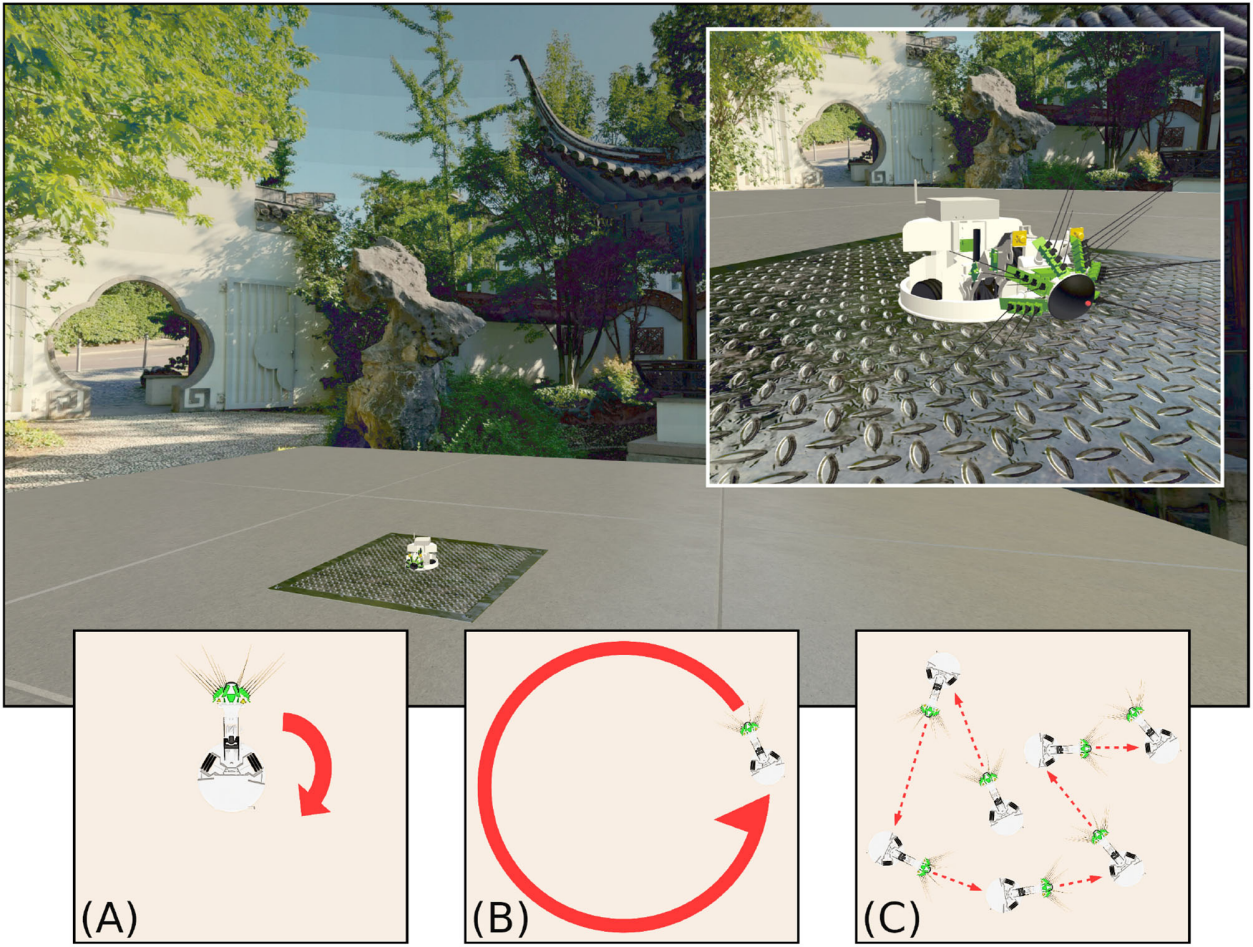
The WhiskEye robot used to capture visual and odometry data sets, as it moved within the simulated environment of the NeuroRobotics Platform. The natural scenery (a panorama of the Chinese Garden in Stuttgart) was projected onto the inside surface of a sphere, surrounding a platform on which WhiskEye can move. The behaviours expressed by WhiskEye during capture of the data sets analysed in this study are referred to as **(A)** rotating, **(B)** circling, and **(C)** random walk.

In models of head direction stabilized by visual input, the visual data used is often contrived. For example, adopting “visual cells” that fire at specific head angles without any real visual data (Song and Wang, 2005) and assume visual processing is performed somewhere upstream. Where true vision is used (captured by cameras), one or more cues positioned in the environment, such as colored panels (Yan et al., 2021) or LEDs (Kreiser et al., 2020), are identified, mapped to a “visual cell,” and learning mechanisms associate this cue with a head angle (Bicanski and Burgess, 2016). Natural visual scenes are much more complex and information rich than bold homogenous cues, with this richness making real-world landmark identification more difficult. The question remains, how does a visual scene become useful for maintaining head angle? In this work we use natural scenes, projected onto a sphere around a simulated robot (see Figure 1. We assume this visual information is distal and invariant to translation. We show that both generative and discriminative model-free learning algorithms can be used to predict head angle from natural visual information and correct for drift in a spiking continuous attractor model of head direction cells, without the need to identify specific landmarks in the environment.

### 1.5. On the Discriminative-Generative Dichotomy

Machine Learning approaches broadly draw from two paradigms. The first is a discriminative paradigm; models which aim to partition the incoming data samples along meaningful boundaries, often building a hierarchy of increasingly abstract representations or increasingly broad sub-spaces, extracting meaning through a bottom-up feedforward process. An example would be Decision Trees, which learn to partition data through recursive splitting on the data space by simple if-else rules (Breiman et al., 1984).

The second is a generative paradigm, which approaches the problem in the opposite direction. A generative model, often also a probabilistic model, aims to instead learn the capability of generating appropriate data samples like the training data in the appropriate contexts. Like a discriminative model, it tries to uncover abstract features in the data, but instead incorporates this into a model of latent features, refining its hypotheses about the underlying causes of the sensory data it is receiving. An example would be Gaussian Mixture Models, which model the problem space as a family of Gaussians with different parameter values (Reynolds, 2009).

Although details between each model vary considerably, the broad trend is that discriminative models are faster than their generative counterparts, but can only work within the bounds of the data they are provided. With the data space's dimensionality being potentially unlimited, this still provides a huge amount of capability, but a training set that does not adequately reflect the data space can lead to nonsensical outputs. Generative models, on the other hand, typically tend to be slower to categorize and slower to learn. However, by generating samples from a model of latent causes of the data, they are not limited by their inputs and can produce very different predictions from the data they are provided. For the case of well-defined and well-bounded problems, this is often surplus to requirements, but for many situations, such as with unfamiliar or incomplete data, this can be beneficial.

Many algorithms make use of elements of both. For example, the Variational Autoencoder (Kingma and Welling, 2014) has hidden layers that extract features from the data in a discriminative way, and use these features to train a multidimensional Gaussian space, the output of which is decoded by another discriminative layer stack to produce a sensible reconstruction of the input. A Generative Adversarial Network (Goodfellow et al., 2014) takes this even further, using a generative and discriminative network in a collaborative competition to produce ever-better data samples. In neuroscience, particularly regarding the visual system, aspects of cortical function have been explained as both a discriminative and generative model, with exactly where and how these approaches synthesize together an active area of research (di Carlo et al., 2021); neural codes originally found in hippocampal work have been hypothesized as a unifying computational principle (Yu et al., 2021); see also Hawkins et al. (2019).

In this study we remain agnostic to the debate, instead choosing to evaluate a mix of generative and discriminative algorithms for generating predictive head direction signals from allothetic (visual) cues. As a purely generative model, a Predictive Coding Network based on MultiPredNet (Pearson et al., 2021), originally from Dora et al. (2018); as a hybrid model, a modification of the JMVAE from Suzuki et al. (2017); and as a purely discriminative model, a Convolutional Neural Network (Lecun and Bengio, 1997).

## 2. Methods

### 2.1. Experimental Apparatus

WhiskEye is a rat-inspired omnidrive robot, with RGB cameras in place of eyes and an array of active whisker-like tactile sensors as shown in Figure 1. In this study only the visual frames from the left camera were considered. A simulated model of WhiskEye was integrated into the Human Brain Project's NeuroRobotics Platform (NRP) as part of prior work (Knowles et al., 2021; Pearson et al., 2021). The NRP integrates robot control and simulation tools, such as ROS and Gazebo, with neural simulators, such as NEST (Falotico et al., 2017). By running these in a synchronized way on a single platform, simulated robots can interact live with simulated neuron models, allowing for experiments with biomimetic and bio-inspired systems. Behaviors can also be specified in more controlled ways using the familiar ROS framework, whilst capturing data from both the robot and neural simulators for off-line analysis.

Using the NRP allows arbitrary visual scenes to be constructed within the environment. The visual scene in this experiment consisted of a concrete-textured floor for WhiskEye to move on; surrounded by an invisible collision mesh to contain the robot in the environment; and with an outer sphere to display the background. The sphere was made large enough so that translation had no perceptible effect on the visual scene; barring the concrete floor, all visual cues could be considered distal. Within this environment, WhiskEye executed three different behaviors: rotating on the spot, circling around the center of the environment and a random walk (illustrated in Figures 1A–C). This provided the odometry and visual data with which to validate the performance of each model.

### 2.2. Spiking Neural Network Model of Head Direction Cell System

The Head Direction system model is a spiking neural network (SNN) model written in pyNEST (2.18; Eppler et al., 2009). All cells are simulated using pyNEST's standard leaky integrate-and-fire neuron model (iaf_psc_alpha) which uses alpha-function shaped synaptic currents. The simulation timestep was set to 0.1 ms for high accuracy with synaptic delay of 0.1 ms. The network is composed of four equally sized rings of neurons: 180 Lateral Mammillary Nuclei (LMN) cells, 180 Dorsal Tegmental Nuclei (DTN) cells, 180 clockwise conjunctive cells and 180 anticlockwise conjunctive cells. Constant input current of 450 pA to all LMN neurons results in spontaneous firing at a rate of 50 spikes per second prior to inhibitory input from the DTN. A summary of the model can be found in Table 1.

**Table 1:** Summary of the spiking neural network written using pyNEST.

	**Model Summary**	
Neuron model	Standard pyNEST Leaky integrate-and-fire neuron model	
Synapse model	*static*_*synapse* does not support any kind of plasticity.	
Plasticity	-	
Topology	Populations arranged as rings	
Measurements	Spikes from LMN population	
	**Populations**	
**Name**	**Size**	**N**
LMN	*N*_*ex*_	180
DTN	*N*_*in*_	180
CW conjunctive	*N*_*ex*_	180
ACW conjunctive	*N*_*ex*_	180
	**Connectivity**	
LMN to DTN	$W_{exc}=b_{exc}exp\;(\frac{1}{2}\frac{-D^{2}}{\sigma^{2}})$ where σ = 0.12 and *b*_*exc*_ = 4000	
DTN to LMN	$W_{inh}=b_{inh}exp\;(\frac{1}{2}\frac{-{\left(D-\mu \right)}^{2}}{\sigma^{2}})$ where σ = 0.12 and μ = 0.5 and *b*_*inh*_ = 450	
LMN to CW conj	One to one, w = 660	
LMN to ACW conj	One to one, w = 660	
CW conj to LMN	*c*[*i*] to *e*[*i*+1], w = 169	
ACW conj to LMN	*c*[*i*] to *e*[*i*−1], w = 169	
	**Input**	
AHV input	One *step*_*current*_*generator* per conjunctive cell population connected to all cells in the population.	
Allothetic input	One *step*_*current*_*generator* per cell in the LMN population connected one to one. Delivers current matching the prediction from the PCN, VAE or CNN.	

**Figure 2:**
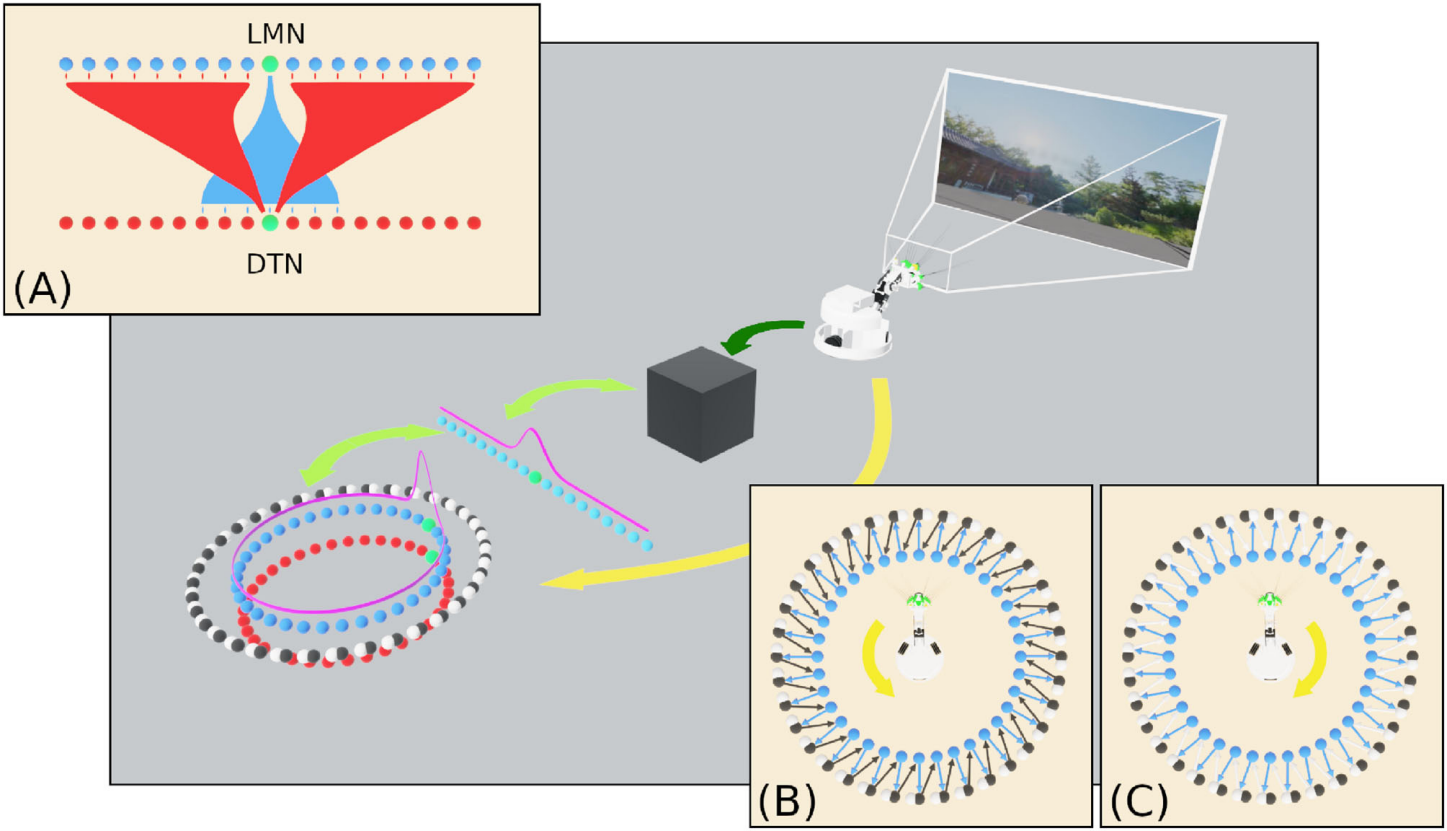
An overview of the SNN model of head direction, the information flows and experimental setup of this study. Visual data from WhiskEye (dark green arrow) and the estimated head angle from the SNN (light green arrows) are used to train one of several model-free learning algorithms (black box). Once trained, these algorithms return head direction predictions (represented as the activity in the array of light blue neurons) that are mapped one-to-one with HD cells in the LMN ring (dark blue neurons) to correct for drift in the spiking HD ring attractor model. **(A)** Excitatory and inhibitory projections between the LMN and DTN respectively for the current most active cell (green neuron). Attractor dynamics emerge from this connectivity to maintain the bump in a stable position in the absence of idiothetic input. **(B)** Connectivity between anticlockwise conjunctive cells (black neurons) and head directions cells offset by one cell anticlockwise. With coincident head direction and angular velocity input (yellow arrow) these cells drive the bump clockwise around the ring. **(C)** Connectivity between clockwise conjunctive cells (white neurons) and head directions cells.

Attractor dynamics emerge through reciprocal connections between cells in the excitatory LMN population and inhibitory DTN population. Each LMN cell *e* connects to a subset of DTN neurons with declining synaptic strength as a function of distance (Figure 2A). Reciprocal inhibitory connections from each DTN cell *i* to LMN cells are arranged with synaptic strength decreasing as a function of distance offset by a constant (μ). This arrangement provides inhibitory input to the cells surrounding the most active LMN cell, producing a single stable bump of activity.

LMN and DTN cells are arranged as rings for the purpose of defining synaptic strength based on distance; this gives the attractor network periodic boundaries. Distances between cells are found (*D*), accounting for the wrap around of the ring. Then synaptic weight from each LMN neuron to DTN neuron (*W*_*exc*_), and return connections from DTN neurons to LMN neurons (*W*_*inh*_) are defined as follows:


$$d1=|\frac{e}{N_{ex}}-\frac{i}{N_{in}}|$$

$$d2=|\frac{e}{N_{ex}}-\frac{i}{N_{in}}-1|$$

$$d3=|\frac{e}{N_{ex}}-\frac{i}{N_{in}}+1|$$

D=min{d1,d2,d3}

$$W_{exc}=b_{exc}exp\left(\frac{1}{2}\frac{-D^{2}}{\sigma^{2}}\right)$$

$$W_{inh}=b_{inh}exp\left(\frac{1}{2}\frac{-{\left(D-\mu \right)}^{2}}{\sigma^{2}}\right)$$

where *e* and *i* are the index of the excitatory and inhibitory cell, respectively. *N*_*ex*_ and *N*_*in*_ are the total number of cells in each ring. *b*_*exc*_ and *b*_*exc*_ are the base weight between the two populations. μ = 0.5 and σ = 0.12, which determine the position of the center of the peak, and the width of the peak, respectively.

In the absence of input from the two conjunctive cell populations, the bump of activity maintained by the attractor network remains stationary. The initial position of the activity bump is produced by applying a 300 pA step current for 100 ms to a nominal LMN cell.

In order to track head direction based on the Angular Head Velocity (AHV) the two populations of conjunctive cells are connected one to one with a LMN cell, shifted one cell clockwise or anticlockwise from the equivalently positioned neuron (Figures 2B,C). Angular velocity of the head was determined by taking the first derivative of the head position captured from the simulated WhiskEye at a rate of 50 Hz, taking the difference in head angle at each time step. Positive values indicated anticlockwise head movements and negative values indicated clockwise head movements. AHV was converted to current (*I*_*V*_) using the following formula:


$$I_{V}=\left(\theta_{t+1}-\theta_{t}\right)\cdot S+I_{min}$$

where θ is the head angle (radians) from the ROS topic published by the robot, *I*_*min*_ = 150 pA, and S = 3500. A step current generator supplies this current to the respective conjunctive cell population. LMN cells also connect one to one with the equivalent conjunctive cell in both the clockwise and anticlockwise populations. Spiking activity occurs in the conjunctive cells with coincident AHV and LMN spiking input. Conjunctive cell input causes movement of the attractor network activity bump to follow head movement.

#### 2.2.1. Allothetic Correction

Head direction predictions from the visual learning models trained on Laplacian shaped representations of head direction (see below), are mapped one to one onto the respective head direction cells. Negative values in predictions are removed by adding the smallest value in the dataset, then prediction values are scaled by a factor of 10 and supplied to the HD network as a direct current injection. This simple method allows predictions which are smaller in magnitude to have less impact on the bump location. However, imprecise predictions, that may have multiple peaks or a broader shape, will lead to current input into more cells compared to a perfectly reconstructed Laplacian.

#### 2.2.2. Analysis of Network Output

To compare the spiking network bump position to the ground truth, the most active cell in each 40 ms time window is found. The difference between the estimated head angle and the ground truth was used to show how accumulation of drift over time, with total error measured as Root Mean Squared Error (RMSE). To illustrate drift in the estimated head angle, the preferred firing direction was plotted using firing rate as a function of head angle in polar tuning curves. The ground truth head direction at each spike time was collected into bins (6°) for the first and the third minute, to show changes in preferred firing direction over time. Differences between idiothetic only and the three correction methods were compared using a one-way ANOVA combined with the Tukey HSD *post-hoc* test. A synthetic set of random uniform predictions, of the same shape and scale as the true predictions, were used to show that reductions in drift were not due to arbitrary current input. Statistical tests were performed using SPSS statistics software.

#### 2.2.3. Artificial Cue Rotation

To investigate the control of allothetic cues over the head direction cell signal, we reproduced cue rotation experiments used in rodent studies (Taube and Burton, 1995). To supply current as if environmental cues were rotated by 90°, the predictions were manipulated by taking either the first 45 or 135 prediction values and shifting them to the end of the 180 element prediction, producing an artificial rotation. This rotation was applied for 30 s after 1 min of standard predictions.

### 2.3. Model-Free Learning Algorithms Applied to Allothetic Cue Recall

#### 2.3.1. Dataset Preprocessing

Each dataset from WhiskEye contained both image data and head direction data. The image data processing was fairly simple, flattening each (width = 80, height = 45) RGB image into a single 10,800 long vector. The head direction data was more involved, being processed as follows:

- Head angle data was recorded at a much higher frequency of 50 Hz rather than the 5 Hz image data. It was therefore subsampled to match the timestamps of the image data.
- Head angle at each timestep from the odometry file was mapped to the 180 cell LMN structure. For example, a head angle of 120° would become a one-hot vector with the max at cell 60.
- In the case of the Spiking Neural Network Estimate, the most active cell in a given 40 ms window was chosen as the active cell for the head direction vector.
- A set of Laplacian distributions was created with means being the active cell of each head direction vector. The Laplacian was chosen over the conventional Gaussian as it lead to better performance overall for the three networks.
- These were rescaled so that the max value for each was 1.

#### 2.3.2. Predictive Coding Network

This network was a modified version of the MultiPredNet (Pearson et al., 2021) which was developed for visuo-tactile place recognition. Here the 3 modules that made up the original network (visual, tactile and multisensory modules) were re-purposed as visual, head-direction and multisensory modules. Compared to the conventional feedforward architectures of other algorithms, the PCN relies on feedback connections toward the input data. For each sample, the PCN outputs a prediction from its latent layer that passes through the nodes of the hidden layers to the input later. The weights between each pair of layers transform the prediction from the upper layer into a prediction of the lower layer's activity. At each layer, the prediction from the layer above is compared to the activity at the current layer and the difference (error) calculated. Weights between layers are then updated locally according to their prediction errors. This eliminates the need for end-to-end backpropagation and increases bio-plausiblity. Several network topologies were trialed; the best performing network had direct odometry input into the multimodal latent layer, hence the lack of hidden layers in that stream (see Table 2 for summary).

**Table 2:** Model parameters and dataset details for PCN, VAE and CNN.

**Parameter**	**Values**		
	**PCN**	**VAE**	**CNN**
Visual input size	10,800	10,800	10,800
Visual hidden layers shape	[1,000, 300]	[1,000, 300]	[32(3,3), 64(3,3)]
Odometry input/output size	180	180	180
MSI layer shape	100	[50, 50]	N/A
Training epochs	200	5,000	50
Full set size	3,000	3,000	3,000
Single set size	390	390	390
SNN estimate size	390	390	390
Test set size	3,000	3,000	3,000
Validation set size	N/A	2,000	2,000
Learning rule	Hebbian	Backprop	Backprop
Optimiser	N/A	SGD	Adam

*PCN training epochs are low due to number of inner ‘cause epochs' (50 for training, 500 for inference) that increase training time, though are strictly a modification of the learning rule rather than extra training epochs. Note that the visual layers for the CNN are 2D Convolution Layers with the kernel shape in brackets. Training epochs vary but all networks were trained to convergence with a single epoch consisting of the entirety of the data for that dataset variant (full, single or SNN estimate). Validation data was taken from a separate set of data gathered with the WhiskEye Rotating behaviour. Hebbian learning is as per Dora et al. (2018), Backpropagation as per Chollet (2015)*.

#### 2.3.3. Multi-Modal Variational Autoencoder

Based on the Suzuki et al. (2017) Joint Multimodal Variational Autoencoder, the VAE works by compressing inputs via hidden layers of decreasing size, encoding inputs into a bifurcated joint multimodal latent space representing the means and variances of Gaussians. These means and variances are used to generate normally distributed random variables, which are then passed through an expanding set of hidden layers to decode the latent Gaussian output into the same shape and structure as the input data. This encoder-decoder system is trained via conventional error backpropagation, comparing the decoded output to the ‘ground truth' input and adjusting weights accordingly, with the addition of a KL-Divergence term to penalize divergence from a μ = 0 Gaussian. As with PCN, the best performing network had no hidden layers between odometry input and the latent layers, so these were removed from both the encoder and decoder halves of the network.

#### 2.3.4. Convolutional Neural Network

As a discriminative network, the CNN handles the task by training its weights so that a given visual input produces the corresponding correct head direction estimate Similarly, unlike the other two networks, the CNN has no latent space to condition and operates purely as a encoder, transforming visual scenes to their appropriate head direction output, with weights updated using conventional backpropagation. It is also the only network that is designed specifically for processing images, with strong spatial priors implicit in the way it processes visual scenes, analyzing small areas of the image in parallel via convolutions to produce translation-invariant image features. As the problem exists within a small, bounded space in both the visual and odometry domains for this experiment, the larger benchmark CNNs—AlexNet (Keshavarzi et al., 2021), ResNets (He et al., 2016) etc.—were not required. Instead, a lightweight, purpose-built CNN was created.

## 3. Results

### 3.1. Head Direction Cell Like Firing Properties

**Figure 3:**
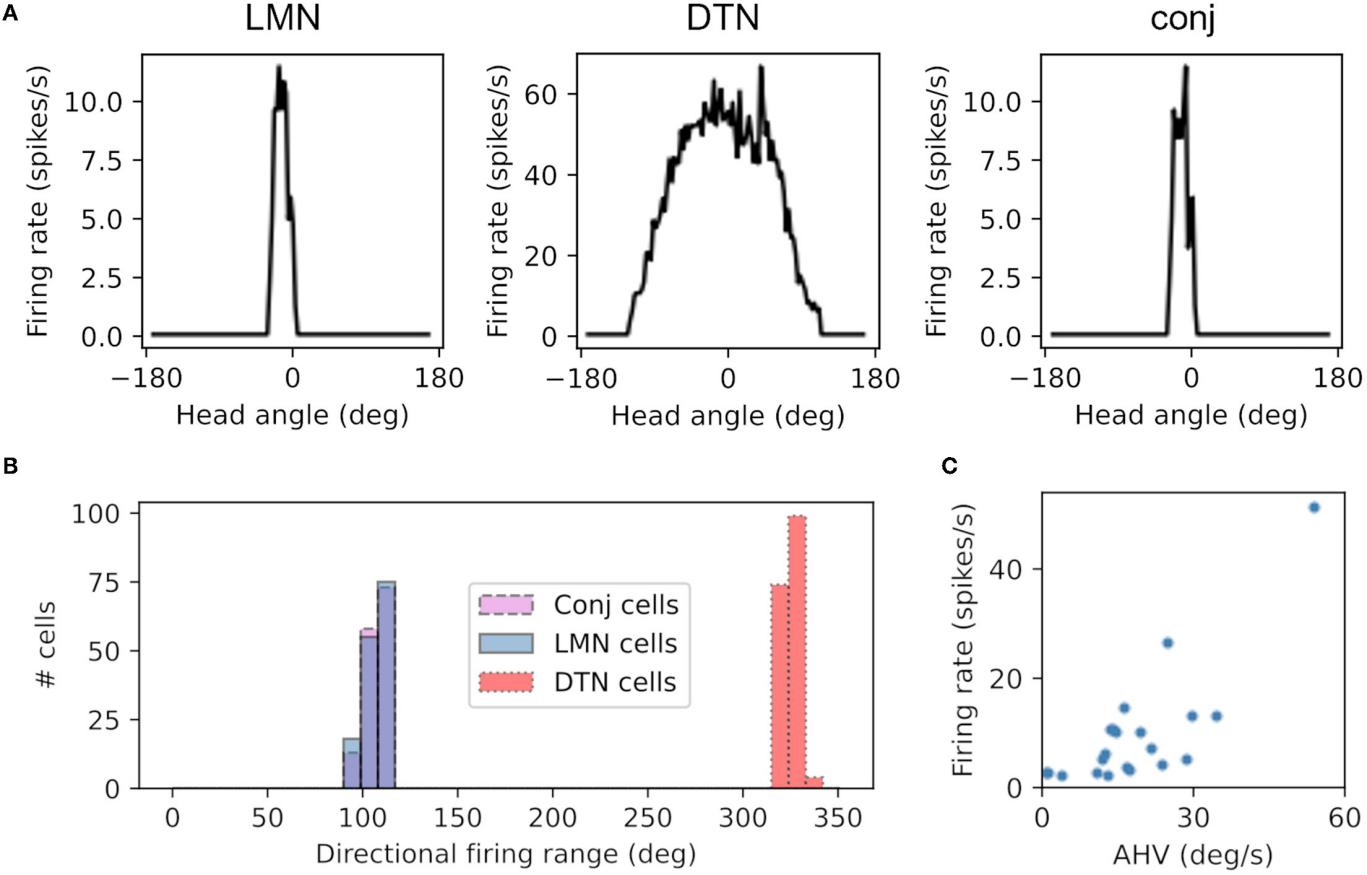
Cells from the DTN, LMN and conjunctive cell populations show HD-like firing characteristics. **(A)** Preferred head angle of one DTN, LMN and conjunctive cell expressed as firing rate as a function of head angle, showing strong directional selectivity in the LMN and conjunctive cell and broader directional selectivity in the DTN cell. **(B)** Histogram of the directional firing range of cells in each population, showing broader directional firing in DTN cells. **(C)** Firing rate as a function of angular head velocity (AHV) from one example conjunctive cell.

Cells in the LMN, DTN and conjunctive cell populations all showed directional firing specificity as observed in the rodent brain. Figure 3A shows firing rate as a function of head direction from the equivalent cell in each of the LMN, DTN and conjunctive cell rings. The preferred firing direction of these cells is taken at the peak firing rate, and the directional firing range is the total range of angles each cell fires over. The average directional firing range of cells in the LMN was 59.3 ± 0.63°, DTN 275.7 ± 0.69° and conjunctive cells 59.2 ± 0.63° (Figure 3B). This is consistent with the directional firing range of DTN head direction cells in rodents (109.43 ± 6.84°; Sharp et al., 2001) being greater than the directional firing range of LMN head direction cells (83.4°; Taube et al., 1990). Figure 3C shows firing rate as a function of angular head velocity for an example conjunctive cell that has similar form to asymmetric AHV cells recorded in the DTN (Bassett and Taube, 2001).

### 3.2. Preferred Firing Direction of Cell Drift With Only Idiothetic Drive

**Figure 4:**
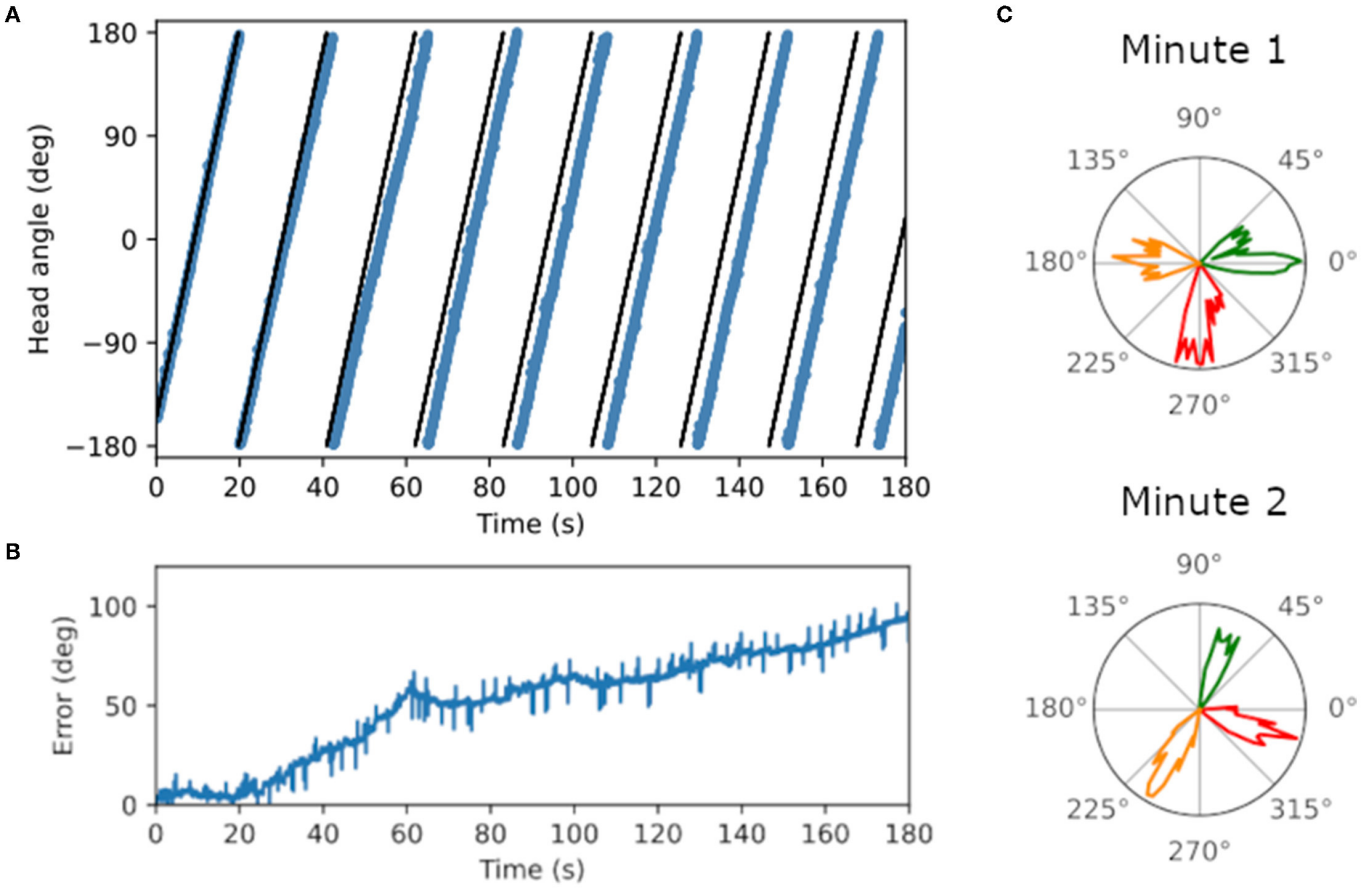
Plots showing drift in the head direction estimate over time. **(A)** Ground truth head angle (black) and the estimated head angle (blue) from the SNN as the WhiskEye rotates on the spot. Over time the estimate gets further from the ground truth. **(B)** Error measured as the magnitude of the difference between the estimated angle and ground truth increases over time. **(C)** Preferred firing directions of three cells (red, orange and green) in the first vs third minute of the simulation, showing a change in preferred firing direction for all three cell of approximately 70°.

Ring attractor dynamics which emerge from reciprocal connections between LMN and DTN cells maintain a stable bump of activity centered on the current estimate of head direction. When movement of the bump is driven only by idiothetic angular velocity input from the two conjunctive cell rings, the preferred direction of head direction cells drifted over time. Figure 4A shows the ground truth (black) and estimated head direction (blue) over time when the WhiskEye robot rotates on the spot. The difference between the ground truth and estimate grows over time (Figure 4B), ending with a maximum difference of 94.5° after 3 min (RMSE = 58.4°). Firing rate as a function of time for 3 LMN cells in the first minute vs the third minute are shown in Figure 4C. The shift in preferred direction of these head direction cells from the first minute to the third minute was 51.3 ± 10.4°. However, the RMSE over the first full revolution was fairly low (5.2°).

### 3.3. Predicting Head Direction Using Model-Free Learning Algorithms

Rodents use allothetic information, such as vision, to counter this drift in head estimate. This requires forming associations between visual scenes and the current head angle, so that the estimated head angle can be corrected when this visual scene is experienced again. Ground truth head direction is not available in biology to form associations between visual scenes and heading. As drift in the head direction estimate (RMSE) is minimal during the first rotation (Figure 4), even when only idiothetic information is available, these early head direction estimates could be used for training the model-free learning algorithms. This would be a much smaller training set; to test the viability of using a such a reduced training set, we first used a single rotation of the ground truth.

This gave us three datasets to train on:

- Full Set - the full 3 min run of ground truth data
- Reduced Set - a single rotation of ground truth data
- SNN Estimate - a single rotation of idiothetic data

**Figure 5:**
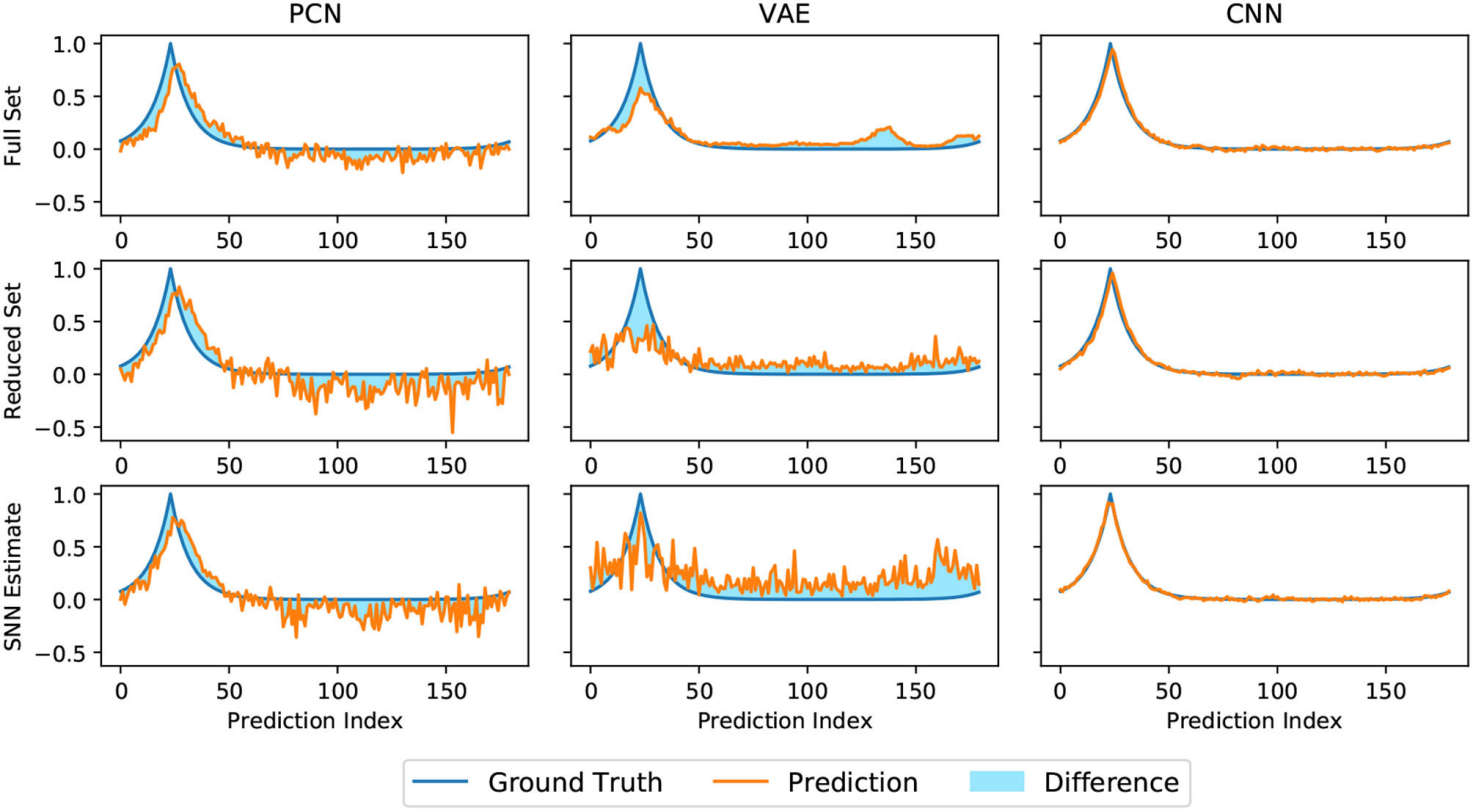
Representative reconstructions of head direction predictions inferred by each algorithm (orange) at a given ground truth head direction (blue). The shaded difference between the two curves illustrates magnitude of the RMSE, which is inversely proportional to the quality of the reconstruction.

Head direction predictions made by three models trained on head direction/vision pairs are not equally structured. As seen in Figure 5, the discriminative CNN is far superior at generating a smooth Laplacian reconstruction, closely approximating the ground truth equivalent for all three variant datasets. The VAE reconstructions consisted of many competing peaks of varying heights, whilst the PCN shows qualities of both, maintaining a Laplacian-esque area of the distribution with noise increasing after a certain distance from ground truth head direction. Both generative models showed a noticeable degradation in the structure of their predictions on the smaller datasets; this is most apparent with the VAE, which suffered further degradation when trained with the SNN estimate.

**Figure 6:**
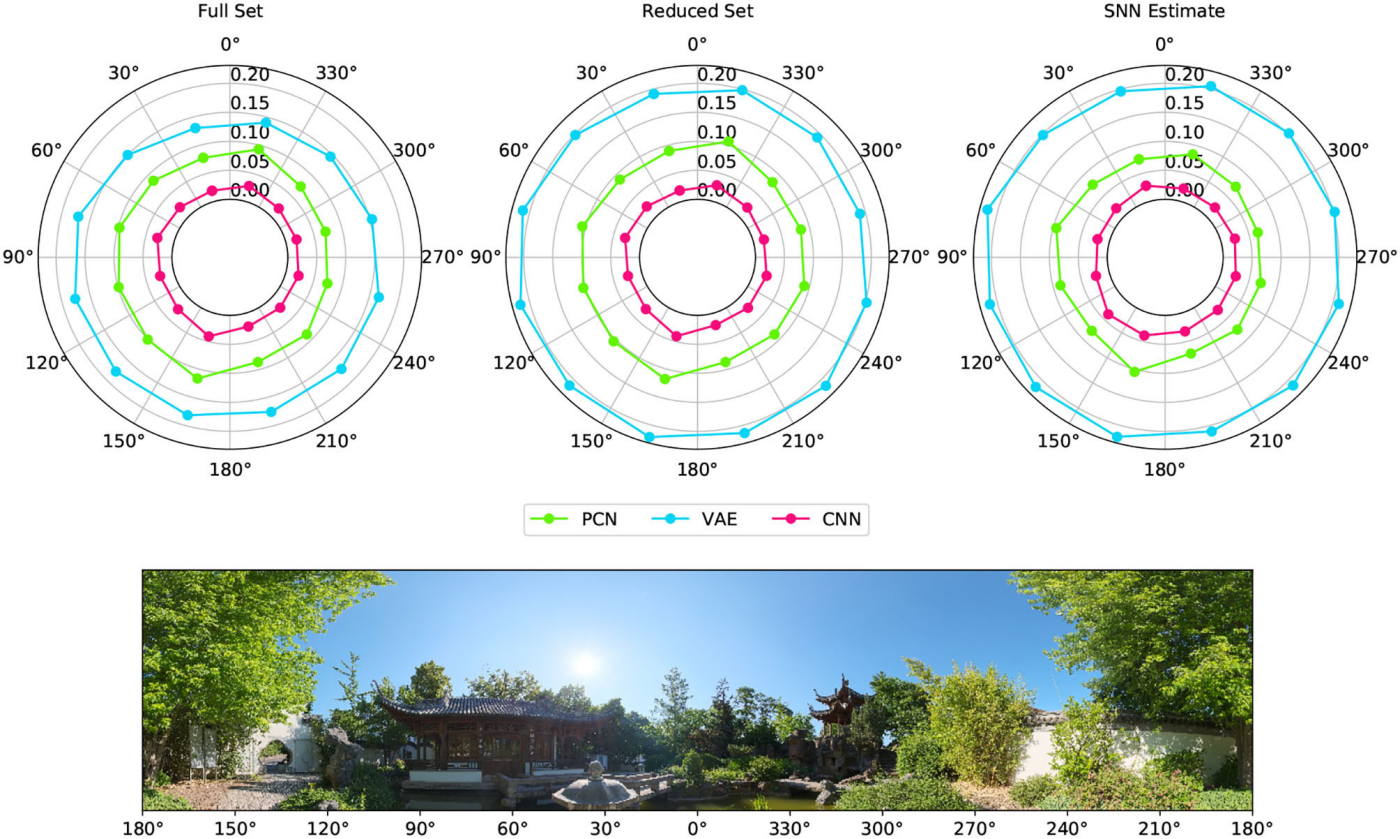
Reconstruction error for different viewpoints of the environment taken from the rotating test dataset. The reconstruction error for each 30° arc of view is represented as a point with radial distance equal to the RMSE between the ground truth Laplacian and each model's reconstruction (PCN, VAE and CNN trained using each of the training sets). The panorama depicts the view of the robot as it rotates on the spot, with associated angular head direction labeled in register with the error polar plots above.

Figure 6 shows the reconstruction error (mean RMSE) for binned views of the visual scene taken from WhiskEye during the rotating behavior. Although there are variations in the error, the performance remains nominally uniform for all head angles, suggesting that the models are not favoring particular features for head direction estimate.

**Figure 7:**
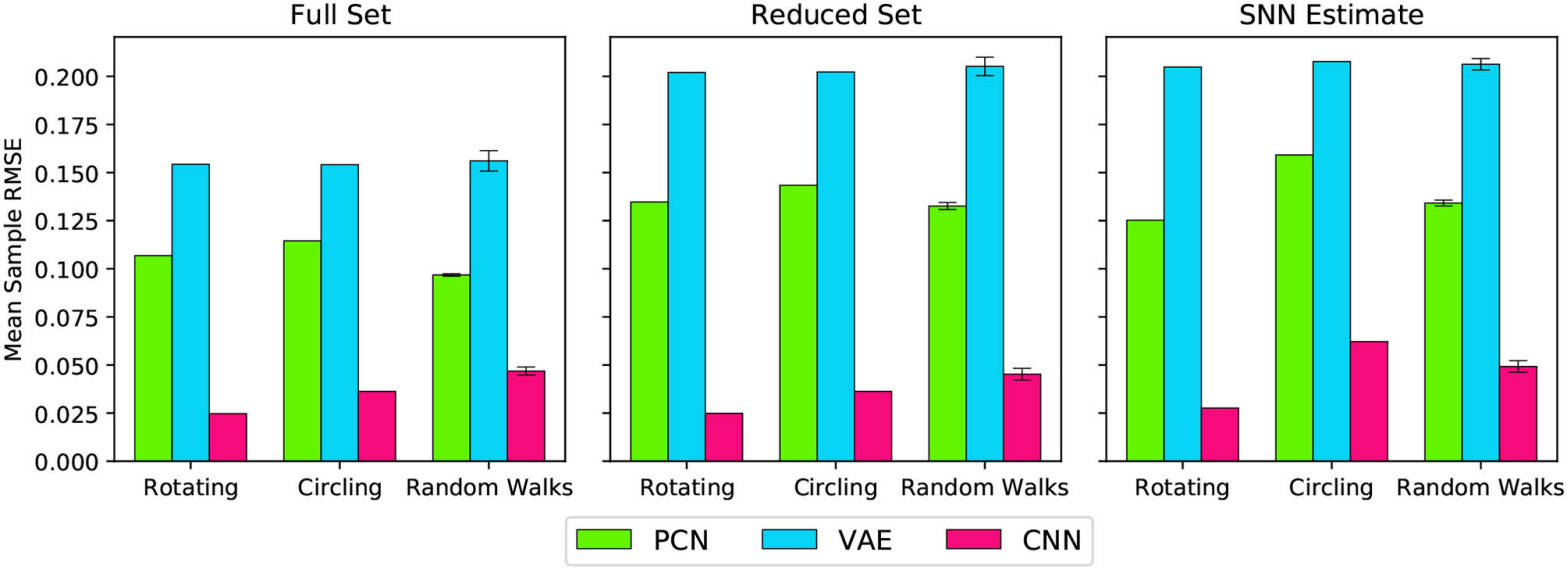
Reconstruction error (RMSE) for each model, during each behaviour, trained on each training set. The Random Walks columns represent the mean of the 5 Random Walk datasets, with error bars indicating the standard error.

Figure 7 shows the overall reconstruction error (mean RMSE) for all datasets and scenarios. For all three models, reconstruction error was noticeably increased by a reduction in dataset quality, but the absolute error remains small. Both the reduced dataset and the SNN estimates were comparable in their error values, demonstrating that the internally generated estimates of the SNN model are a suitable substitute for ground truth odometry as a teaching signal, provided the dataset (and therefore accumulated drift) is small. Further to this, it demonstrates the effectiveness of all three methods, and thus their representative paradigms, at performing this task with limited data.

### 3.4. Drift Reduction Using Head Direction Predictions as Allothetic Input

**Figure 8:**
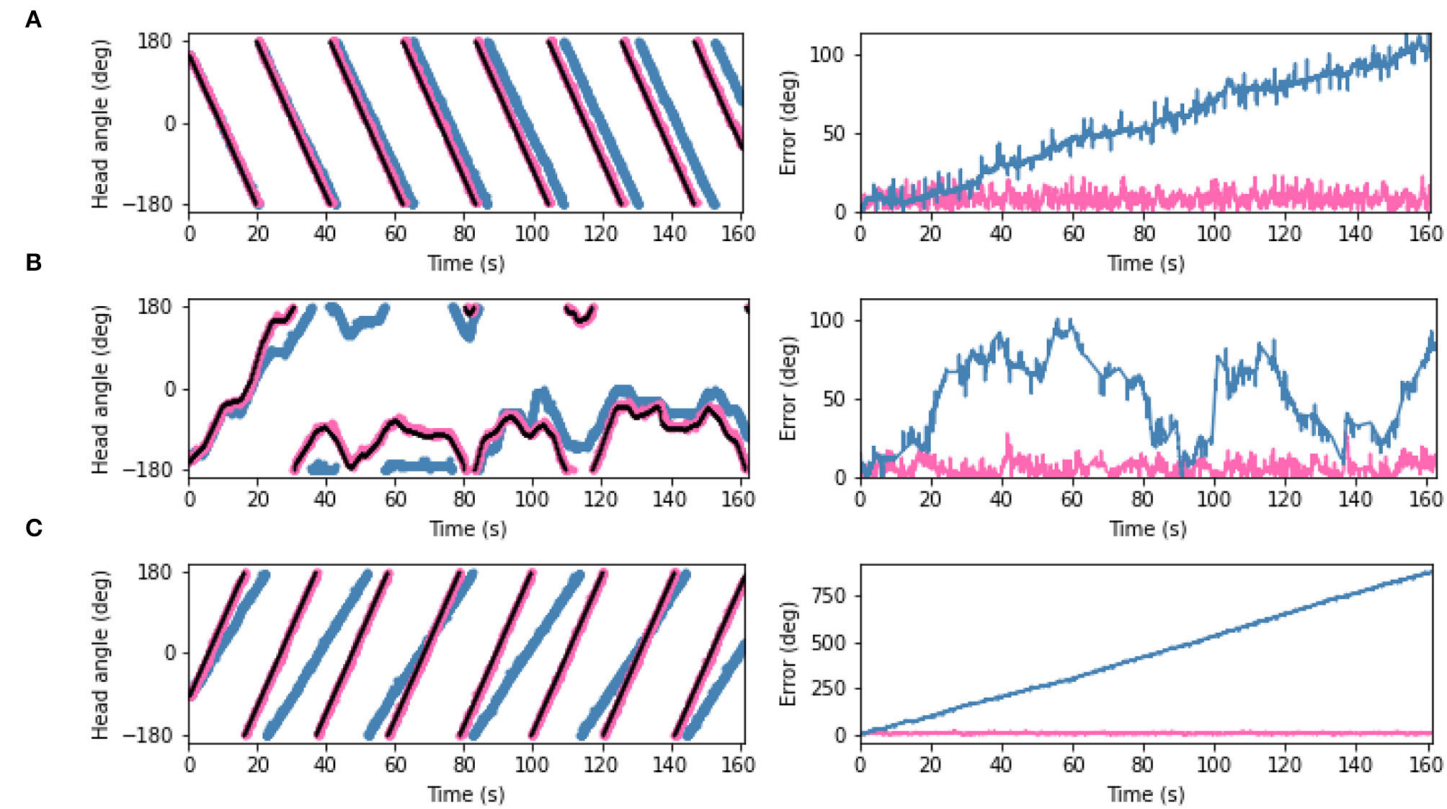
(Left) Plots showing estimated head angle from the SNN with idiothetic drive only (blue), the corrected estimated head angle from the SNN which also receives allothetic input from the PCN (pink), and ground truth head angle (black). (Right) The difference between each estimate and the ground truth as also shown. Examples are shown from the **(A)** rotating, **(B)** random walk 1, and **(C)** circling datasets. In all cases, the allothetic correction results in minimised drift and the corrected estimate and ground truth are almost indistiguishable. As the PCN, VAE and CNN produce similar reductions in drift, only the PCN plots are shown.

The predictions generated by the PCN, VAE, and CNN trained on the full dataset were converted into one-to-one current inputs to LMN cells to correct for drift using visual information. Figure 8 shows the ground truth head direction, idiothetic only estimate and the corrected estimate for 3 example datasets (rotation, a random walk, and circling), with the respective error over time. In each case, the corrected head direction estimate (pink) is much closer to the ground truth (black) than the estimate using idiothetic input only (blue), which drifts over time. Across all five random walk datasets, corrective input from the PCN, VAE and CNN all significantly reduced drift (one way ANOVA with Tukey HSD *post-hoc* testing: PCN *p* = 0.001; VAE *p* < 0.001; CNN *p* < 0.001, Table 3). The smallest error after corrections was achieved using predictions made by the CNN, which had the lowest reconstruction error. Even though VAE predictions are imprecise, it still performs comparably to the other methods. This may be due to current inputs onto HD cells far from the active bump having less influence due to the attractor dynamics; only current inputs close to the bump location have strong influence over bump position. Although the drift was large for the circling dataset (RMSE = 558.6°), all three methods successfully corrected for this drift. This was the biggest reduction in error for all three model-free learning algorithms (difference in RMSE: PCN 549.5°, VAE 555.2°, CNN 556.3°).

**Table 3:** RMSE (degrees) of the difference between the estimated head direction from the model and the ground truth using only idiothetic drive, and with corrections from the PCN, VAE or CNN trained on each of the three training sets.

		**Full set RMSE (°)**			**Reduced set RMSE (°)**			**SNN estimate RMSE (°)**		
	**Ideo only**	**PCN**	**VAE**	**CNN**	**PCN**	**VAE**	**CNN**	**PCN**	**VAE**	**CNN**
Rotation	69.64°	9.41°	3.44°	2.68°	9.41°	8.35°	2.31°	6.73°	6.99°	2.71°
Circling	558.81°	9.58°	3.72°	2.24°	14.21°	9.44°	2.47°	14.21°	14.86°	2.49°
Random 1	63.33°	7.02°	5.47°	4.24°	8.64°	15.70°	4.32°	9.02°	15.98°	3.22°
Random 2	58.18°	6.39°	4.71°	4.17°	8.73°	10.74°	4.64°	9.03°	18.97°	3.73°
Random 3	126.90°	7.25°	6.35°	2.61°	9.27°	18.47°	2.88°	10.21°	16.50°	4.05°
Random 4	203.09°	16.83°	13.21°	12.34°	14.36°	18.49°	10.62°	14.89°	16.83°	12.34°
Random 5	65.03°	7.09°	5.91°	3.35°	8.72°	12.08°	3.49°	8.42°	15.83°	3.57°

### 3.5. Drift Reduction Using a Reduced Training Set

The PCN, VAE, and CNN were trained using a single rotation of ground truth head directions, and the same method used to convert the predictions into current input to the head direction cells. In all cases, the RMSE between ground truth and the estimate head direction was reduced. Across all five random walk datasets, corrective input from the PCN, VAE and CNN all significantly reduced drift (one way ANOVA with Tukey HSD *post-hoc* testing: PCN *p* = 0.001; VAE *p* = 0.002; CNN *p* < 0.001, Table 3). Once again the largest error reductions were achieved using CNN predictions. The VAE corrections were the least helpful, reflecting the larger reconstruction error when training on the reduced dataset.

### 3.6. Drift Reduction Using SNN Estimate as Training Set

As the reduction in drift was comparable when the full 3 min ground truth and a single revolution were used as training sets, we trained each of the model-free learning algorithms on a single revolution of the estimated head direction produced by the spiking model.

**Figure 9:**
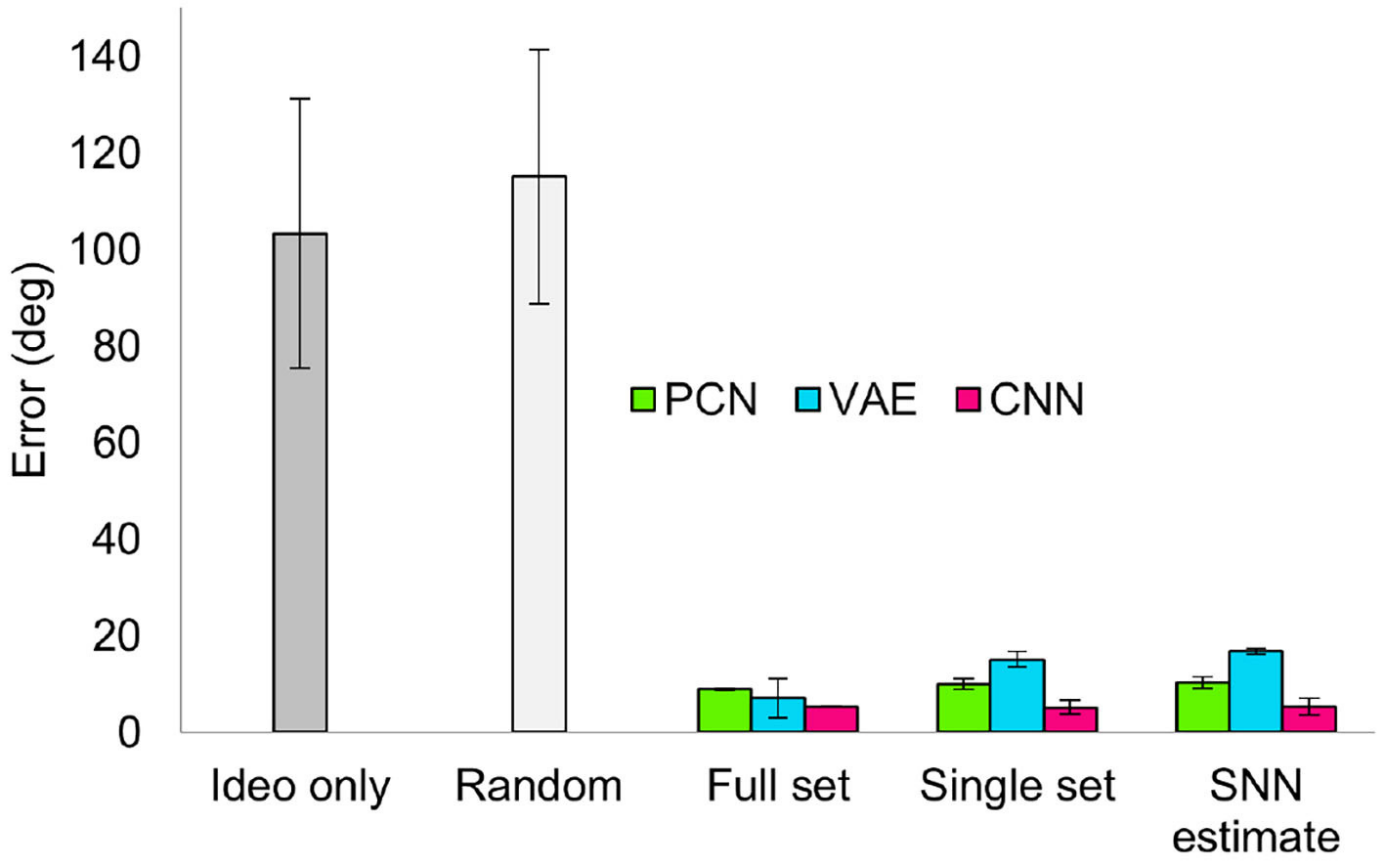
Summary of drift reduction for each of the model-free learning algorithms and training sets across all 5 random walk datasets. Compared to idiothetic input only and random predictions, drift is significantly reduced for all three methods trained on full, single and SNN estimate training sets. Bar plot shows average error (degrees) ± standard error.

Similar to the drift reduction seen for the previous two training sets, drift was reduced by all three models trained on each of the datasets. Across all five random walk datasets, corrective input from the PCN, VAE and CNN all significantly reduced drift (one way ANOVA with Tukey HSD *post-hoc* testing: PCN *p* = 0.001; VAE *p* = 0.002; CNN *p* < 0.001, Table 3). The CNN produced the best error reduction, ahead of the PCN and then the VAE, reflecting the reconstruction error of their predictions. With each decrease in training set quality from full ground truth, first to single revolution ground truth and then to single revolution estimated head direction, the average error across the random walks increased for both the PCN and VAE, remaining stable only for the CNN. Figure 9 shows a summary of drift reduction by all three model-free learning algorithms trained on the full, reduced and SNN estimate training sets. Compared to head direction estimates which rely only on idiothetic input, or randomly generated predictions, all methods and training sets produced a significant reduction in drift.

### 3.7. Cue Rotation

**Figure 10:**
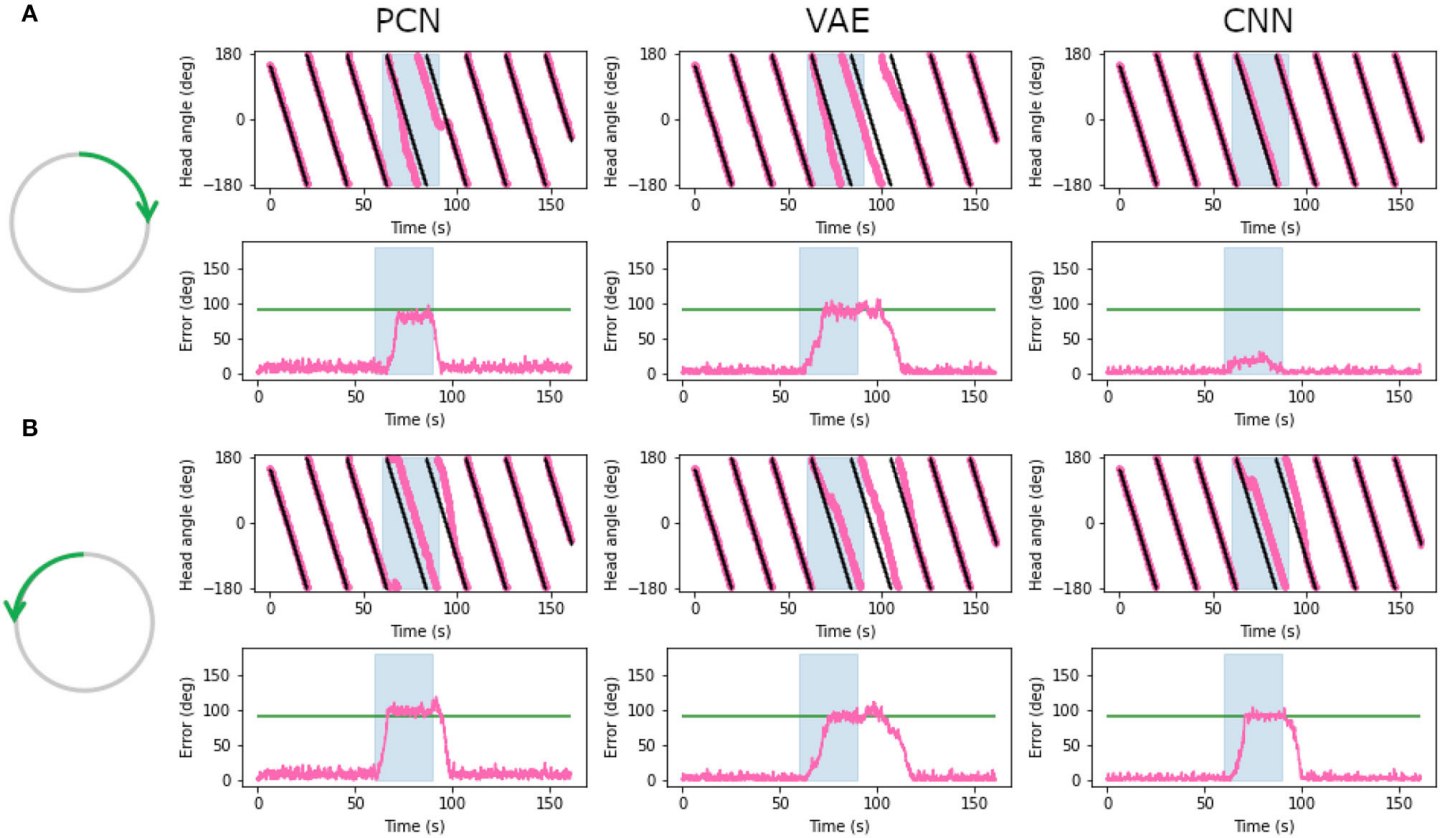
Plots showing corrected estimated head angle (pink) compared the ground truth (black) during the artificial cue rotation experiments. The blue block indicates the period of cue rotation either clockwise **(A)** or anticlockwise **(B)**; the expected rotation (90°) is indicated with a green line on the error plot. In both clockwise and anticlockwise rotations, corrections by the PCN and the VAE move the bump to the rotated position after a delay. The CNN fails to pull the bump contrary to the direction of bump movement.

Head direction cells in rodents have been shown to follow environmental cues over their idiothetic estimate of heading, even when those cues are rotated within the environment (Taube and Burton, 1995; Yoder et al., 2015). To replicate a cue rotation experiment using the WhiskEye rotating behavior, we provided unaltered allothetic predictions from each of the model-free learning algorithms for the first minute, then rotated 90° either clockwise or anticlockwise for 30 s before returning to unaltered allothetic predictions. Figure 10 shows the head direction estimate against the ground truth, with the error for clockwise (Figure 10A) and anti-clockwise (Figure 10B) rotations of the allothetic input from the PCN, VAE and CNN trained on the full ground truth. The green line shows an offset of 90°, which is the rotation of the cue and the value the error is expected to reach.

For both clockwise and anti-clockwise rotations, the PCN and VAE input strongly control the bump position. After a short delay, the bump position moves the full 90°, error between ground truth and the estimate reaching the green line. When the rotation is removed the bump continues to follow the allothetic input after a delay. Some drift may be required before the allothetic input can gain control over the bump position, resulting in a delay. In the case of the CNN, only when the idiothetic drive and the rotation were in the same direction (Figure 10B) could the allothetic input control the bump position strongly enough to complete the full rotation. Because the allothetic and idiothetic input are provided simultaneously, the bump is more likely to move when both of these pull the bump in the same direction around the ring rather than compete with each other.

The CNN has consistently the lowest reconstruction error of all three methods (Figure 5), producing predictions with a sharp Laplacian peak. This prediction shape results in current input to a small number of cells at a precise position, and produces the most accurate head direction estimate. This is likely because the amount of drift between each allothetic correction is small, and the bump does not need to be moved far. Noisier predictions from the VAE and PCN result in current injection to more cells, making it less accurate for drift correction but more able to move the bump large distances, as in this cue conflict case. These data suggest a refined Laplacian peak is not the most effective prediction shape for strong allothetic control over the head direction estimate. In all cases, the current magnitude used was high enough to correct for drift without impairing idiothetic control. By varying the amount of current supplied, allothetic input could have stronger or weaker control over the bump position regardless of prediction shape.

## 4. Discussion

With these experiments we have shown that, like head direction cells recorded in rodents, a spiking continuous attractor model of head direction cells driven purely by self-motion (idiothetic) information is subject to drift. Taking inspiration from a number of previous studies (Boucheny et al., 2005; Song and Wang, 2005; Shipston-Sharman et al., 2016), we exploit reciprocal excitatory and inhibitory connections between the LMN and DTN to produce attractor dynamics which maintain a bump of activity at the estimated head angle.

Drift is thought to be caused by imprecise self-motion cues, but may also be due to inaccuracies in the model of angular head velocity (AHV). Variability within environments or the body, such as injury (or in robots; inaccuracy in the odometry data due to wheel slip), make maintaining a precise model of AHV at all times unlikely. A prominent limitation of the experimental apparatus is that the odometry from the robot being collected from a simulated embodiment is not subject to inaccuracies. However, the stochastic nature of a spiking model limits the resolution and range of angular velocities which can be accurately represented by a single neuron, this can be seen clearly in the large drift accrued during the circling dataset where head angle changes very slowly. Using a population code rather than single cells may allow for a finer resolution of AHVs which can be represented in spikes, and contribute to reducing drift.

In rodents, drift in the preferred firing directions of head direction cells is seen mainly in the dark or when brain regions providing allothetic input are lesioned (primarily visual; Yoder et al. 2015), indicating these data are essential for stabilizing the head direction signal. Using predictions from three different model-free learning algorithms, we directly influenced the bump position, minimizing drift. In some previous models of drift correction, allothetic information contributes to calibrating the model of AHV, rather than using allothetic input to directly change the bump position. Kreiser et al. (2020) refine the AHV model by detecting error between the estimated head angle and learnt positions of landmarks, and altering firing properties of AHV cells. Stratton et al. (2011) suggest a role for specific behaviour patterns for learning new landmarks and calibrating the AHV model. We show that predictions made after training on the estimated head angle from the SNN during a single revolution—a specific behavior—can be used to successfully correct for drift.

Entrainment of the head direction signal to visual information has been seen in cue rotation studies, where external environmental cues are rotated in the environment and a corresponding rotation is observed in the preferred firing direction of the head direction cells. These large changes in bump location are better solved by influencing the bump position directly, rather than updating the AHV model. By rotating the allothetic predictions, we have replicated shifts in the bump position to match the rotation of the environment. As AHV cell firing also shows some refinement when visual information is available (Keshavarzi et al., 2021), going forward a combination of optimizing the AHV model and direct bump movement could be used.

A Laplacian-shaped input centered on the current HD was used to train the three model-free learning algorithms. The CNN reproduced this shape in its prediction whereas the VAE and PCN produced broader, more Gaussian-like predictions. The CNN consistently produced the most precise head direction predictions even for the small SNN estimate training set. This suggests a trivial learning problem for the CNN, likely because the range of possible distal views observed by the robot is small and bounded; the same frames used for training are likely to be reobserved as the robot rotates. However, even with less precise predictions the PCN and VAE can reduce drift significantly, likely due to the attractor dynamics dampening current inputs far from the bump location. The artificial cue conflict experiments revealed a precise Laplacian distribution not to be suitable as a corrective signal due to the limited number of cells current is injected into, and therefore the limited power of this input to influence the bump location. In contrast, the broader predictions made by the PCN and the VAE were able to better control the bump position; refining the shape and strength of the prediction would likely change the allothetic control over the bump. Two populations of head direction cell have been identified in rodents, those more controlled by allothetic input and others more strongly controlled by idiothetic input (Dudchenko et al., 2019); we can see how by varying the strength or shape of allothetic input to the network, these two cell types may emerge.

In previous work, correcting drift with the aid of visual information has either assumed visual processing upstream and provided correction based on the ground truth (Song and Wang, 2005), or learnt the orientation of arbitrary features, such as LEDs or colored panels (Kreiser et al., 2020; Yan et al., 2021). Here we show that corrective signals can be generated by learning associations between natural visual scenes and a self generated representation of heading, without identifying specific environmental landmarks. However, we recognize that including advanced visual processing and feature extraction may be useful for online learning mechanisms to determine the reliability of visual input. This type of corrective allothetic signal is presumed to come from the postsubiculum; lesions of this region lead to more drift than seen for control animals in the dark (Yoder et al., 2015). This suggests that this region may be contributing more than just visual correction, but also other sensory modalities. In this paper, we have focused on the calibration of head direction estimate by visual inputs; an intriguing direction for future work would be the inclusion of other allothetic information, such as tactile or olfactory. In visually ambiguous environments, conflicting visual cues may cause the HD estimate to become less accurate. Olfaction has great potential for detecting loop closures, as rodents leave scent trails as the explore environments (Peden and Timberlake, 1990), which can tell them if and how long ago they visited a position. A recent study in mice has shown that blind animals can use olfactory information to correct for drift in the head direction estimate (Asumbisa et al., 2022).

All of the methods in this paper currently require batch learning of head direction-image pairs, however, as rodents continue to move within environments, they must continually learn and refine associations between head angle and visual scenes. Learning to place less weight on unreliable cues, such as the position of the sun, which may initially appear as a useful landmark but becomes unstable with time (Knight et al., 2014), is key to reliable correction of head angle in dynamic natural environments. The next step is to adapt these model-free learning algorithms to learn continuously and adapt their predictions as the robot explores its environment.

The three trained models, despite their differences in reconstruction error, are all good candidates for generating allothetic corrections for the SNN. Although some scenarios such as cue conflicts show weakness of overly-precise estimates as by the CNN, this is not a fault of the model itself; the robustness of the PCN and VAE predictions to cue conflicts shows that learning to minimize the RMSE from a Laplacian ground truth signal is not ideal for the task at hand, and that better performance could be gained by training to a broader distribution (such as a Gaussian).

Where differences do lie is in their applicability to more complex experimental setups. The environment the data is gathered from is simple in structure despite the complexity of the visual scene; there are no proximal cues to obscure the environment and sensory input is limited to vision. Previous studies have shown non- visual and multimodal examples of CNNs (Ma et al., 2015; Dauphin et al., 2017) and VAE architectures (Suzuki et al., 2017) can perform well. Both, however, have issues with scaling: the multimodal CNN requiring many stacked networks working together, and multimodal VAEs requiring many intermediate uni-modal latent spaces to perform the task successfully. It is an open question as to how well PCNs will scale into more than 2 modalities and whether they will run into similar scaling issues as the VAEs. However, its method of operation and learning rule are bio-plausible, with local learning making it the best candidate for implementation as a spiking model. Furthermore, prior work has already shown that a PCN can use tactile information to inform localization (Pearson et al., 2021).

This work has raised many important questions. How robust are these model-free learning approaches to a changing world, particularly with multiple environments, visually and potentially tactually distinct from each other? How can these be trained in a sequential manner, as an animal would experience them, whilst avoiding the catastrophic forgetting of earlier environments? Can the bio-plausibility of this system be increased by making the learning fully online, and is the SNN estimate of head direction reliable for long enough to train one of these algorithms to produce useful corrections? The experimental apparatus developed and used in this study are well placed to address these questions.

## 5. Conclusion

Idiothetic control of the head direction system is especially important in new, ambiguous or dark environments; allothetic control increases the accuracy of the head direction estimate and may help refine idiothetic control or make large corrections after a period of drift. We have shown that natural visual scenes, without identifying specific landmarks, can be used to predict the current head angle by training three model-free learning algorithms; this on a limited and imprecise training set of estimated head angles, produced by a spiking continuous attractor model of the head direction cell system, driven by idiothetic inputs from robot odometry. Predictions from all three methods were equally valuable in minimizing drift.

## Data Availability Statement

The datasets generated for this study, along with code for the NEST and Machine Learning models, can be found in the HeadDirectionPredNet repository: https://github.com/TomKnowles1994/HeadDirectionPredNet.

## Author Contributions

RS built the spiking neural network model. TK built the simulation experiments and gathered datasets. All authors contributed to the article and approved the submitted version.

## Conflict of Interest

The authors declare that the research was conducted in the absence of any commercial or financial relationships that could be construed as a potential conflict of interest.

## Publisher's Note

All claims expressed in this article are solely those of the authors and do not necessarily represent those of their affiliated organizations, or those of the publisher, the editors and the reviewers. Any product that may be evaluated in this article, or claim that may be made by its manufacturer, is not guaranteed or endorsed by the publisher.
